# Serum tumor markers combined with HRCT for malignancy risk assessment of solitary pulmonary nodules: a retrospective study

**DOI:** 10.3389/fgene.2026.1731395

**Published:** 2026-04-01

**Authors:** Yong Zhou, Shuitang Wang, Yongqiu Zhang

**Affiliations:** 1 Imaging Diagnosis Center, The 3rd Affiliated Teaching Hospital of Xinjiang Medical University (Affiliated Cancer Hospital), Urumqi, Xinjiang, China; 2 Department of Respiratory Medicine, The 905th Hospital of PLA Navy, Shanghai, China; 3 Department of Radiology, The 905th Hospital of PLA Navy, Shanghai, China

**Keywords:** CEA, CYFRA 21-1, high-resolution CT, lung cancer, NSE, solitary pulmonary nodules

## Abstract

**Introduction:**

This study aims to investigate the correlation between serum tumor markers (CEA, NSE, CA-125, and CYFRA 21-1) and imaging findings in patients with solitary pulmonary nodules, and to assess their value in predicting the risk of malignancy.

**Methods:**

A retrospective analysis was conducted on 110 patients with solitary pulmonary nodules, of whom 45 were benign and 65 were malignant. The clinical data, serum tumor marker levels, CT imaging findings, and diagnostic efficacy of single and combined tests were compared between the two groups.

**Results:**

Serum levels of CEA, CA-125, CYFRA 21-1, and NSE in the malignant nodule group were significantly higher than those in the benign nodule group (P < 0.001). CT imaging revealed that patients with malignant nodules typically exhibited characteristics such as deep lobulation, pleural indentation, short fine spiculation, and multiple cystic lucencies, whereas the benign nodule group more commonly displayed pleural thickening and satellite lesions. The diagnostic efficacy of combined CT and tumor marker testing was significantly superior to that of single tests, with a sensitivity of 96.9% and an accuracy of 87.3%. The area under the curve (AUC) of the combined detection was significantly higher than that of any single indicator (P < 0.05).

**Discussion:**

The combined detection of serum tumor markers and high-resolution CT imaging findings has high clinical value in the diagnosis of benign and malignant solitary pulmonary nodules, offering a more precise basis for cancer risk assessment.

## 1 Introduction

Lung cancer is one of the most prevalent malignant tumors worldwide and remains the leading cause of cancer-related mortality (Jemal et al., 2025; Barta et al., 2019). In 2020, it was reported that there were approximately 2,206,771 new cases of lung cancer were diagnosed globally, accounting for 11.4% of all cancer diagnoses, and 1,796,144 deaths, representing 18.0% of all cancer fatalities, and that lung cancer remains the leading cause of cancer-related deaths and the most commonly diagnosed cancer worldwide (Zheng et al., 2022). Early lung cancer often presents as a solitary pulmonary nodule (SPN), characterized by a well-defined lesion with a diameter not exceeding 3 cm, surrounded by aerated lung tissue, and without associated atelectasis, hilar enlargement, or pleural effusion (Harzheim et al., 2015; Chan et al., 2017).

SPN is usually asymptomatic and can be either benign or malignant, which makes early differential diagnosis difficult and leads to low detection rates. Therefore, early identification of its nature and individualized treatment are crucial to the health of patients.

With the advancement of imaging technology, high-resolution computed tomography (HRCT) offers significant advantages over conventional CT in displaying detailed pulmonary lesions (Zhou et al., 2024). However, due to the variability of SPNs, HRCT still presents limitations in diagnostic accuracy. Moreover, imaging examinations are constrained by the subjectivity of post-processing techniques and the experience of the examiner, which makes it difficult to achieve complete accuracy and objectivity. In recent years, serum tumor markers, as a non-invasive diagnostic tool, have gradually become an important auxiliary means for differentiating benign from malignant pulmonary nodules (Xu et al., 2024).

Among numerous serum tumor markers, carcinoembryonic antigen (CEA) is relatively common in lung adenocarcinoma, and its level is correlated with tumor burden and invasiveness. Cytokeratin 19 fragment (CYFRA 21-1) is considered an important marker for non-small cell lung cancer, especially squamous cell carcinoma. Neuron-specific enolase (NSE) is closely related to small cell lung cancer and neuroendocrine differentiation. Although carbohydrate antigen 125 (CA125) is mainly used for ovarian cancer screening, studies have found that it may also be elevated in lung cancer, especially in patients with pleural involvement or adenocarcinoma subtypes. Therefore, these biomarkers reflect the heterogeneity of lung cancer from different pathological types and biological characteristics.

This study hypothesizes that the combination of serum tumor markers (CEA, CA-125, CYFRA 21-1, and NSE) and HRCT imaging features can significantly improve the diagnostic performance in distinguishing benign from malignant SPNs and in assessing the risk of malignancy, compared with single detection. Based on this, the present study aims to investigate the clinical value of this combined method for the differential diagnosis and risk assessment of SPNs. The innovation of this study lies in combining multiple serum tumor markers with different diagnostic specificities with HRCT imaging features to employ a combined detection strategy, which is then validated by pathological results, thereby providing a more accurate reference for the clinical risk assessment of SPNs.

## 2 Materials and methods

### 2.1 Case selection

A retrospective analysis was conducted on the clinical data of 110 patients with SPNs treated at our hospital from January 2022 to June 2024, including 55 males and 55 females, aged 32–79 years, with an average age of (55.95 ± 1.79) years. The nodules varied in size from 6 to 30 mm, with an average diameter of (17.75 ± 1.65) mm. Through chest CT imaging, serum tumor marker testing, and clinical follow-up, a total of 110 patients were included, comprising 45 benign cases and 65 malignant cases. The current study was approved by the 905th Hospital of PLA Navy Ethics Committee. All patients and their families agreed to participate in the experiment and signed the informed consent form. Inclusion criteria: (1) Complete and comprehensive examination results of imaging and tumor markers and clinical data; (2) Imaging results indicating a SPN with a maximum diameter ≤30 mm, well-defined lesion, and surrounded by aerated lung tissue, in accordance with the diagnostic criteria for SPNs; (3) Confirmation through pathological tissue examination or needle biopsy cytology; (4) Benign nodules confirmed through follow-up for at least 15 months after surgery or biopsy; (5) First-time detection of the tumor or nodule, with no prior history of surgery, radiofrequency ablation, radiotherapy, or chemotherapy. Exclusion criteria: (1) Patients with underlying diseases such as lung, liver, kidney, or heart conditions; (2) Patients with a previous diagnosis of lung cancer who had received relevant treatment; (3) Patients with other types of malignant tumors or diseases affecting the lungs; (4) Pregnant or breastfeeding women; (5) Incomplete pathological data.

### 2.2 Methods

#### 2.2.1 Tumor marker detection

Fasting venous blood samples (5 mL) was collected from all subjects in the early morning. After natural coagulation, the blood was centrifuged at 3,000 rpm for 10 min. The serum was then separated and stored for later use. The concentrations of CEA, NSE, CYFRA 21-1, and CA125 in the serum were measured using the Roche Cobas e602 automated immunoassay analyzer (Roche Diagnostics GmbH, Germany), with the testing process strictly adhering to the operational protocols of the equipment and reagent kits. Diagnostic criteria were as follows: CEA >5 ng/mL, NSE >16.3 ng/mL, CYFRA 21-1 > 3.3 ng/mL, and CA125 > 24 kU/L were considered positive. A parallel testing strategy was adopted for the combined detection, in which the result was considered positive if HRCT or any serum tumor marker yielded a positive finding. This approach was designed to improve the detection rate of malignant nodules.

#### 2.2.2 CT scan

The Discovery CT750 HD CT scanner with GSI (GE Healthcare, USA) was used, and routine CT and HRCT scans were performed preoperatively. (1) Routine CT scan: A continuous scan of the patient’s entire lung field was conducted, employing standard algorithms for reconstruction, with slice thickness and spacing both set to 5 mm. (2) HRCT scan: A targeted scan was performed on the lesion site, with reconstruction slice thickness and spacing set to 0.6 mm, utilizing high-resolution algorithms for reconstruction. According to the specific circumstances, post-processing techniques such as two-dimensional and three-dimensional reconstruction, along with minimum density projection were applied to present the lesion characteristics from multiple angles and comprehensive perspectives. The CT images were analyzed by two senior radiologists using a double-blind method, with particular attention given to imaging features indicative of malignancy, such as the presence of vacuole sign, lobulation, spiculations, pleural indentation, and vascular convergence. Through a comprehensive analysis, a determination was made regarding the benign or malignant nature of the lesion. In cases of disagreement, consensus was reached through negotiation. Immunohistochemical staining results were used as the pathological reference standard to validate CT imaging findings and their diagnostic performance in assessing the risk of malignancy in SPNs, thereby establishing a comprehensive diagnostic approach combining CT imaging and pathology.

### 2.3 Statistical analysis

Data analysis was conducted using the SPSS 23.0 statistical software. Continuous data were first tested for normality using Shapiro–Wilk test. Data conforming to a normal distribution were compared between groups using the independent-samples t-test, whereas non-normally distributed data were analyzed using the Mann–Whitney U test. Continuous data were presented as mean ± standard deviation (SD). Categorical data were analyzed using chi-square tests, with results presented as the number of cases and percentage (n, %). Intergroup comparisons were performed using Z-tests. P < 0.05 was considered statistically significant.

## 3 Results

### 3.1 Comparison of clinical data

This study included 110 patients with SPNs, of whom 45 had benign nodules and 65 had malignant nodules. No statistically significant differences were observed between the benign and malignant nodules groups in terms of gender, smoking history, family history of tumors, and nodule density distribution (P > 0.05). However, significant differences were found between the two groups in terms of age distribution, nodule diameter, and location (P < 0.05). Notably, the malignant nodule group had a higher proportion of patients aged ≥65 years, with nodule diameters between 20 mm and 30 mm, and nodules located in the right upper lobe (Table 1).

**TABLE 1:** Comparison of clinical data.

Clinical data	Benign nodule group (n = 45)	Malignant nodule group (n = 65)	*χ* ^*2*^ */t*	*P*
Gender				
Male	24	31	0.339	0.561
Female	21	34		
Age (years)				
<65	27	24	5.695	0.017
≥65	18	41		
Smoking history	29	34	1.601	0.206
Family history of tumors	10	14	0.007	0.932
BMI (kg/m^2^)	23.5 ± 1.7	23.2 ± 1.4	1.012	0.314
Nodule diameter (cm)				
<10 mm	9	6	7.546	0.023
11 mm–19 mm	25	27		
20 mm–30 mm	11	32		
Nodule density				
Ground-glass nodules	11	8	2.747	0.253
Part-solid nodules	8	13		
Solid nodules	26	44		
Nodule location				
Upper lobe of the right lung	7	28	9.669	0.022
Middle lobe of the right lung	5	5		
Lower lobe of the right lung	13	15		
Upper lobe of the left lung	7	12		
Lower lobe of the left lung	13	5		

### 3.2 Comparison of serum tumor marker levels

**FIGURE 1:**
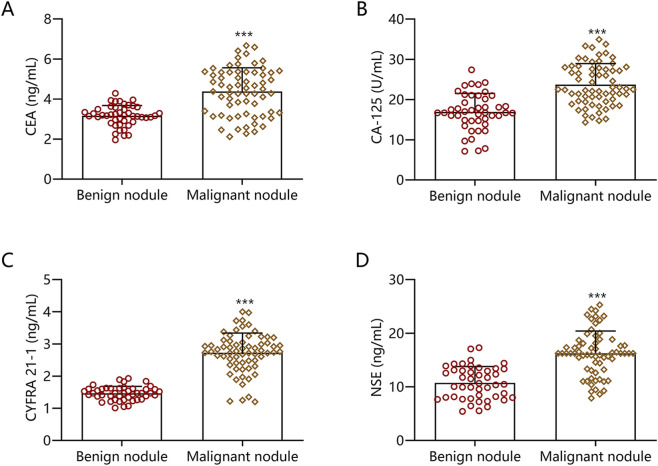
Comparison of Serum Tumor Marker Levels in Benign and Malignant SPNs. **(A)** CEA; **(B)** CA-125; **(C)** CYFRA 21-1; **(D)** NSE. Note: Compared with malignant nodules, ***P < 0.001.

Serum levels of CEA, CA-125, CYFRA 21-1, and NSE in the malignant nodule group were significantly higher than those in the benign nodule group, with statistically significant differences (P < 0.001) (Figure 1).

### 3.3 Comparison of CT signs of pulmonary nodules

In the malignant nodule group, the detection rate of typical nodules with deep lobulation, pleural indentation, short fine spiculation, multiple cystic lucencies, bronchial vascular convergence, and spiculate protuberance, whereas the benign nodule group exhibited higher detection rate of adjacent pleural thickening and satellite lesions. These differences were statistically significant (P < 0.05) (Table 2).

**TABLE 2:** Comparison of CT signs of benign and malignant pulmonary nodules.

CT signs	Benign pulmonary nodules (n = 45)	Malignant pulmonary nodule (n = 65)	*χ* ^*2*^	*P*
Typical nodules with deep lobulation	4 (8.89)	30 (46.15)	17.291	<0.001
Pleural indentation	3 (6.67)	24 (36.92)	15.219	<0.001
Short fine spiculation	4 (8.89)	28 (43.08)	15.067	<0.001
Multiple cystic lucencies	2 (4.44)	14 (21.54)	6.251	0.012
Bronchial vascular convergence	2 (4.44)	12 (18.56)	4.704	0.030
Spiculate protuberance	5 (11.11)	23 (35.38)	8.257	0.004
Adjacent pleural thickening	10 (22.22)	4 (6.15)	6.181	0.013
Satellite lesions	16 (35.56)	2 (3.08)	20.495	<0.001

### 3.4 CT imaging characteristics of pulmonary nodules of different natures

**FIGURE 2:**
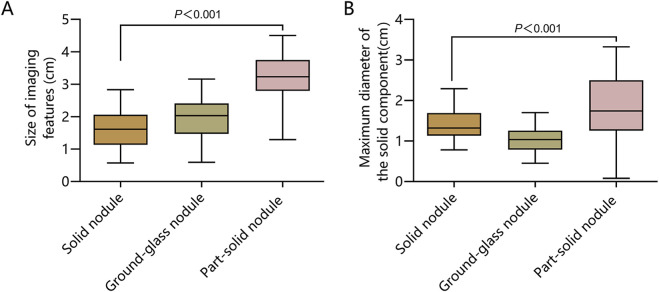
CT Imaging Characteristics of Pulmonary Nodules of Different Natures. **(A)** Size of CT imaging characteristics; **(B)** The maximum diameter of the solid component.

The size of CT imaging characteristics and the proportion of the maximum diameter of the solid component in patients with pulmonary nodules of different natures revealed statistically significant differences (P < 0.05), as shown in Figure 2.

### 3.5 Diagnostic efficacy of CT and tumor markers in single and combined tests

Using pathological examination as the gold standard, the sensitivity, specificity, and accuracy of CT, tumor markers, and their combined use in detecting malignant tumors were evaluated. The results showed that pathological diagnosis confirmed 65 cases of malignant tumors (100%). Among these, HRCT identified 50 cases (76.9%), tumor markers detected 36 cases (55.4%), and the combined test identified 63 cases (96.9%) (Table 3). The differences in sensitivity among the three methods were statistically significant (P < 0.05). The combined test exhibited the highest sensitivity, but the lowest specificity; in terms of accuracy, tumor marker testing had the lowest accuracy, while the combined test had the highest accuracy. CT showed the highest positive predictive value, while tumor marker testing had the lowest negative predictive value (Table 4).

**TABLE 3:** Comparison of diagnostic results between the single and combined tests.

CT	Pathological examination			Tumor markers	Pathological examination			Combined detection	Pathological examination		
	Positive	Negative	Total		Positive	Negative	Total		Positive	Negative	Total
Positive	50	5	55	Positive	36	7	43	Positive	63	12	75
Negative	15	40	55	Negative	29	38	67	Negative	2	33	35
Total	65	45	110	​	65	45	110	​	65	45	110
*χ* ^2^	6.722			*χ* ^2^	12.250			*χ* ^2^	5.786		
P	0.010			P	0.001			P	0.016		

Serum tumor markers used in this study included CEA, CA-125, CYFRA, 21-1, and NSE.

**TABLE 4:** Comparison of sensitivity, specificity and diagnostic efficacy of single and combined tests.

Indicators	Sensitivity	Specificity	Accuracy	Positive predictive value	Negative predictive value
CT scan	76.9%	88.9%	81.8%	90.9%	72.7%
Serum tumor markers	55.4%	84.4%	67.3%	83.7%	56.7%
Combined test	96.9%	73.3%	87.3%	84.0%	94.3%

Serum tumor marker tests in this study included CEA, CA-125, CYFRA, 21-1, and NSE.

### 3.6 Diagnostic value of single and combined detections in diagnosing the benign and malignant nature of SPNs

**FIGURE 3:**
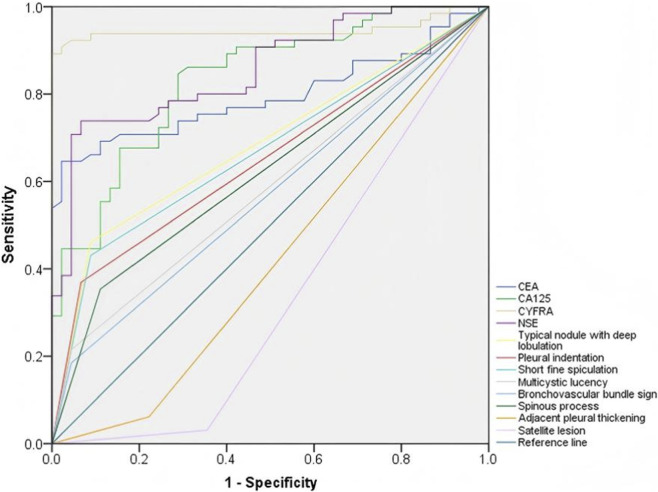
AUC for the combined diagnosis of benign and malignant SPNs by HRCT and tumor markers.

The results of this study revealed that the combined use of CEA, CA-125, CYFRA 21-1, and NSE along with HRCT significantly enhanced the predictive accuracy for determining the benign or malignant nature of SPNs, as evidenced by a markedly higher area under the curve (AUC) compared to each individual marker. The AUC for the combined detection strategy exceeded 0.95, indicating its substantial clinical utility, with a statistically significant difference (P < 0.05) (Table 5; Figure 3).

**TABLE 5:** Value of single and combined detections in differential diagnosis of benign and malignant SPNs.

Indicators	AUC	*SE*	*P*	*95% CI*
CEA	0.797	0.043	<0.001	0.711∼0.880
CA125	0.834	0.038	<0.001	0.759∼0.908
CYFRA	0.946	0.025	<0.001	0.896∼0.996
NSE	0.856	0.035	<0.001	0.787∼0.925
Typical nodules with deep lobulation	0.686	0.050	0.001	0.588∼0.785
Pleural indentation	0.651	0.052	0.007	0.550∼0.753
Short fine spiculation	0.671	0.051	0.002	0.571∼0.771
Multiple cystic lucencies	0.585	0.054	0.129	0.479∼0.691
Bronchial vascular convergence	0.570	0.055	0.213	0.463∼0.677
Spiculate protuberance	0.621	0.053	0.031	0.517∼0.726
Adjacent pleural thickening	0.420	0.057	0.153	0.309∼0.531
Satellite lesions	0.338	0.055	0.004	0.229∼0.446
Combined	0.950	0.019	<0.001	0.912∼0.987

### 3.7 Analysis of representative case images

**FIGURE 4:**
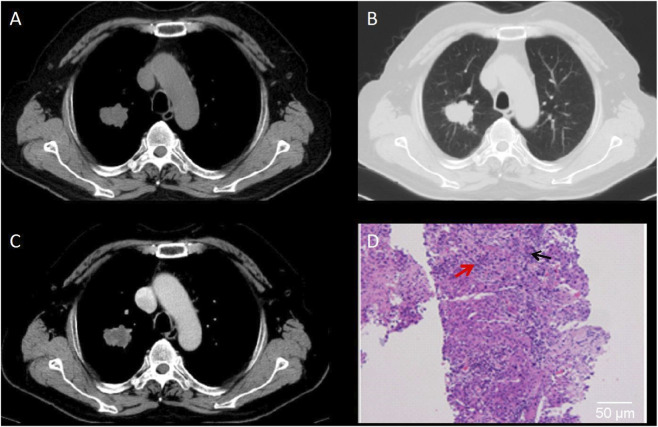
CT Images of Invasive Non-Mucinous Adenocarcinoma. **(A)** CT mediastinal window shows a round-to-oval nodule in the right upper lobe with irregular margins. **(B)** CT lung window reveals a lobulated nodule with spiculated margins. **(C)** Contrast-enhanced CT scan demonstrates an uneven moderate enhancement of the lesion. **(D)** Histopathological examination shows invasive lung adenocarcinoma; hematoxylin-eosin staining, ×200 magnification. Black arrow indicates densely arranged tumor cells with infiltrative growth. Red arrow indicates marked nuclear atypia and hyperchromasia. Scale bar = 50 μm.

**FIGURE 5:**
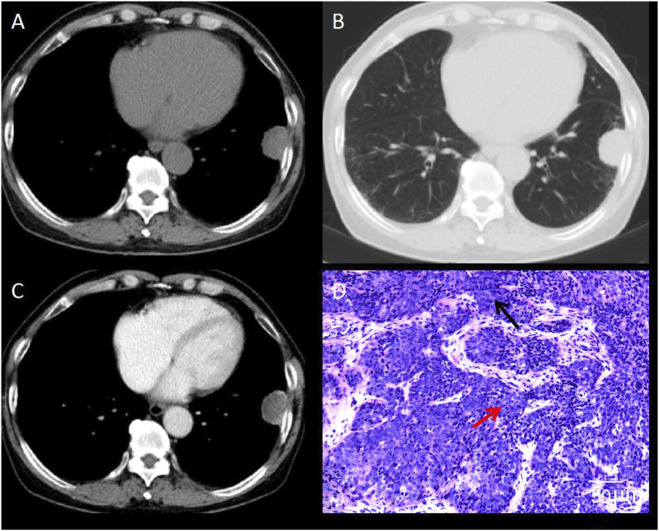
CT Images of Poorly Differentiated Squamous Cell Carcinoma. **(A)** CT mediastinal window shows a round-to-oval solid nodule adjacent to the pleura in the right lower lobe, with relatively well-defined margins. **(B)** CT Lung window reveals a solid nodule adjacent to the pleura. **(C)** Contrast-enhanced CT scan demonstrates moderate homogeneous enhancement of the lesion. **(D)** Histopathological examination reveals poorly differentiated squamous cell carcinoma (hematoxylin-eosin staining, ×200 magnification). Black arrow indicates irregular glandular structure with infiltrative growth. Red arrow indicates marked nuclear atypia and hyperchromasia. Scale bar = 50 μm.

**FIGURE 6:**
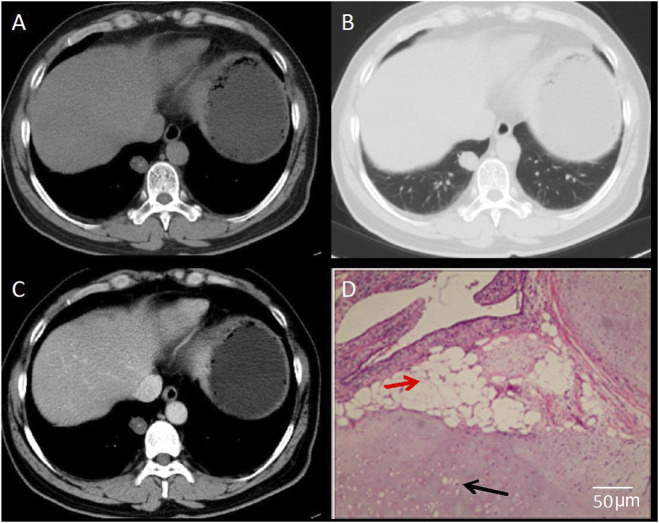
CT Images of Pulmonary Hamartoma (Cartilage Type). **(A)** CT mediastinal window shows a round-to-oval nodule adjacent to the pleura in the right lower lobe, with well-defined margins. **(B)** CT lung window demonstrates a solid nodule adjacent to the pleura. **(C)** Contrast-enhanced CT scan reveals minimal enhancement of the lesion. **(D)** Histopathological examination confirms pulmonary hamartoma (hematoxylin-eosin staining, ×100 magnification). Red arrow indicates mature adipose tissue. Black arrow indicates cartilaginous tissue. Scale bar = 50 μm.

**FIGURE 7:**
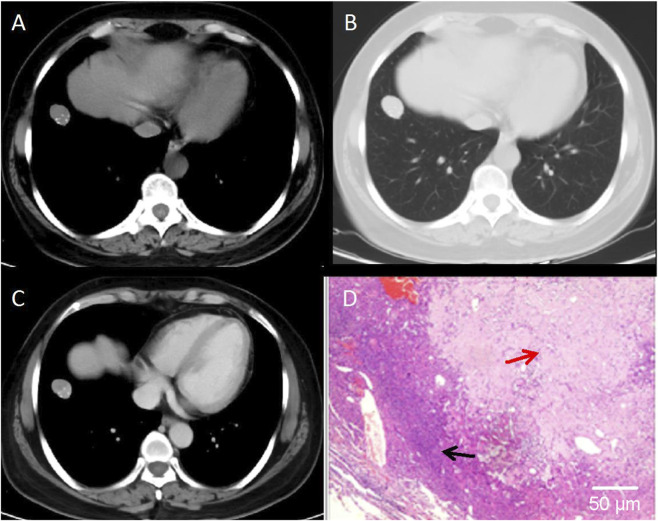
CT Images of Sclerosing Pneumocytoma **(A)** CT mediastinal window shows a round-to-oval nodule in the right lower lobe with relatively high density. **(B)** CT lung window demonstrates a solid nodule with well-defined margins. **(C)** Contrast-enhanced CT scan reveals minimal enhancement of the lesion. **(D)** Histopathological examination confirms pulmonary sclerosing pneumocytoma (hematoxylin-eosin staining, ×100 magnification). Black arrow indicates densely cellular areas. Red arrow indicates necrosis. Scale bar = 50 μm.

This study combined CT imaging findings with immunohistochemical staining results to perform a comprehensive analysis of different types of pulmonary nodules, so as to validate the diagnostic value of CT in assessing the risk of malignancy in SPNs. The results revealed that Figure 4 demonstrates a lobulated nodule in the right upper lobe with spiculation, showing moderate, uneven enhancement on contrast-enhanced scans. The pathological diagnosis confirmed it as invasive non-mucinous adenocarcinoma. Figure 5 depicts a nodule in the left lower lobe with short spiculation at the margins and moderate enhancement on contrast-enhanced scans. The pathology identified it as poorly differentiated squamous cell carcinoma. Figure 6 illustrates a nodule in the right lower lobe with well-defined borders, calcific foci, and fat density, with no significant enhancement on contrast scans. The pathology was identified as a pulmonary hamartoma (cartilage type). Figure 7 also shows a nodule in the right lower lobe with uniform density and calcific foci, exhibiting marked uniform enhancement on contrast scans, and the final pathological diagnosis was sclerosing pneumocytoma. The imaging features are consistent with the pathological findings, underscoring the critical role of imaging in the diagnosis of pulmonary nodules (Figures 4–7).

## 4 Discussion

According to statistics, approximately 30%–50% of SPNs are ultimately confirmed to be malignant tumors (Chen et al., 2024), with some patients missing critical treatment opportunities due to delayed diagnosis. Therefore, for SPN patients, rapid and accurate differentiation of the lesion nature is crucial for treatment and improving prognosis. This study aims to evaluate the diagnostic value of serum tumor markers combined with HRCT imaging features in distinguishing benign from malignant SPNs and assessing the risk of malignancy. The results showed that the serum levels of CEA, CA-125, CYFRA21-1, and NSE in patients with malignant pulmonary nodules were significantly higher than those in patients with benign nodules, and their HRCT imaging showed distinct malignant characteristics. Further analysis revealed that the combined testing significantly outperformed the single detection in terms of sensitivity, accuracy, and AUC, validating the objectives and hypotheses of this study.

Previous studies (MacMahon et al., 2005) have demonstrated that the risk of lung cancer significantly increases with age, with the malignancy rate in patients over 65 years of age exceeding 85%, 2.25 times higher than in younger patients. In this study, malignant SPNs accounted for 36.9% of cases in patients under 65 years of age, and 63.1% in those aged 65 and above, which is consistent with the aforementioned findings. Additionally, smoking is a major risk factor for lung cancer, with malignant pulmonary nodules growing more rapidly in smokers, and the disease more likely to progress to advanced stages (Swensen et al., 1999). Pulmonary nodules are found in almost all smokers over the age of 50 at first screening, with approximately 10% of them developing new nodules within a year (Larsen and Minna, 2011). However, in this study, no significant differences were observed between benign and malignant SPN cases in terms of gender, smoking history, or family history. This may be attributed to the relatively small sample size, which does not completely rule out the potential correlation of these factors with malignant SPN. Future research should expand the sample size, incorporate additional variables, and conduct multicenter studies to further explore the epidemiological characteristics of SPN. In addition, the findings of this study indicated that the highest proportion of SPNs occurred in the right upper lobe, with malignant SPNs in the right upper lobe accounting for 43.1%, which is consistent with previous studies. Research (Takahashi et al., 2010) indicates that among 360 SPN patients with pathological results, approximately 37.5% of pulmonary nodules are located in the right upper lobe, and about 41.4% of malignant SPNs smaller than 20 mm are found in the right upper lobe. This suggests that the distribution of SPN nodules plays a significant role in distinguishing between benign and malignant lesions. Furthermore, among nodules of varying densities, part-solid nodules have the highest likelihood of malignancy, followed by solid nodules and ground-glass nodules (Ye et al., 2024). The distribution proportions of nodules with varying densities showed no statistically significant differences between benign and malignant nodules, which may be attributed to the small sample size; further studies with a larger sample are needed for more comprehensive observation.

CEA is highly expressed in malignant tumors of the gastrointestinal tract and pancreas (Jansen et al., 2024), and elevated CEA in SPN may suggest an increased likelihood of lung cancer (Xue et al., 2024). However, CEA lacks specificity and may also be elevated in other malignant tumors or benign conditions, thus limiting its utility as a sole diagnostic marker. CA125 is primarily utilized for ovarian cancer screening and monitoring; however, it may also be elevated in cases of pulmonary metastatic ovarian cancer (Barlow et al., 2024). Therefore, elevated CA-125 levels in SPNs reflect an increased overall risk of malignancy rather than being highly specific for lung cancer. CYFRA 21-1, a fragment of keratin 19, is closely linked to non-small cell lung cancer, particularly squamous cell carcinoma (Xu et al., 2024). An elevated level of CYFRA 21-1 indicates an increased likelihood of malignancy and exhibits relatively high diagnostic specificity among serum markers for lung cancer. NSE, an enzyme associated with neuronal and neuroendocrine cells, is commonly elevated in neuroendocrine neoplasm such as small cell lung cancer (Zhu et al., 2024). In patients with SPNs, elevated NSE levels may indicate a neuroendocrine origin of the nodule, with particular attention required for the possibility of small cell lung cancer (Liu et al., 2024). Based on the differences in specificity and complementary characteristics of the above-mentioned biomarkers in different pathological types of lung cancer, this study selected the combined detection of CEA, CA-125, CYFRA 21-1, and NSE to address the issue of insufficient specificity in single indicators. The results of this study showed that the levels of CEA, CA-125, CYFRA 21-1, and NSE in patients with malignant pulmonary nodules were significantly higher than those in patients with benign pulmonary nodules, consistent with previous findings. This further supports the clinical value of the combined use of serum tumor markers in distinguishing benign from malignant SPNs and assessing the risk of malignancy, serving as an important auxiliary diagnostic method for imaging examinations.

HRCT offers superior spatial resolution compared to conventional CT. Through thin-slice scanning and precise reconstruction, it minimizes artifact interference and provides a clear depiction of lesion characteristics. By combining two-dimensional target reconstruction with three-dimensional imaging techniques, it enables multi-angle analysis of the relationship between lesions and surrounding structures, thereby significantly enhancing diagnostic accuracy. Common signs on HRCT include lobulation, vacuole sign, vascular convergence, and pleural indentation (Wang et al., 2018). Vacuole sign manifests as small focal lucent areas or low-density shadows within the lesion, typically resulting from residual lung tissue or bronchiectasis, and is a significant early sign of lung cancer. Lobulation sign refers to the arcuate protrusions or fissured lobes at the margins of a nodule, associated with the growth rate and differentiation variations of the lesion. Spiculation sign manifests as short radial shadows on the border of the lesion, often resulting from cancerous infiltration, whereas benign spiculation is generally caused by fibrosis or inflammatory exudation, often accompanied by pleural adhesions or thickening. Pleural indentation sign appears as a conical image between the lesion and the pleura, more commonly seen in malignant lesions, though some benign conditions, such as tuberculosis, may also present this feature, linked to local inflammatory reactions. Bronchial vascular convergence sign refers to cancerous tissue invading the bronchovascular bundle or interlobular septum, resulting in the retraction of surrounding structures, with a higher occurrence in malignant nodules than in benign ones. CT imaging reveals vascular thickening, displacement, and direct connection to the lesion, with some regions exhibiting umbilical-like indentation (Xue et al., 2024). This study revealed that patients with malignant pulmonary nodules exhibited typical malignant features, such as lobulation, spiculation, and pleural indentation, with a significantly higher frequency than benign pulmonary nodules. These radiological characteristics are consistent with previous studies, particularly the presence of deep lobulation and short fine spiculation, which show a markedly higher detection rate in malignant nodules compared to benign ones, suggesting that detailed imaging has high diagnostic value in distinguishing between benign and malignant lesions.

While CT exhibits a relatively high specificity (88.9%), its sensitivity remains comparatively low (76.9%). On the other hand, although serum tumor markers demonstrate a higher sensitivity in the early detection of lung cancer, their specificity is relatively low (84.4%). Combined testing can overcome the limitations of individual methods, significantly enhancing both diagnostic sensitivity and accuracy (with a combined sensitivity of 96.9% and accuracy of 87.3%). Further analysis revealed that the combined use of HRCT and serum tumor markers yielded a significantly higher AUC than each individual marker, with an AUC exceeding 0.95. This indicates that the combined detection strategy holds promising clinical prospect. Notably, in patients with indeterminate pulmonary nodules, this combined diagnostic approach effectively enhances early diagnostic accuracy, providing a more reliable foundation for subsequent treatment decisions. This study further validated the consistency between imaging examinations and pathological diagnoses through the analysis of typical cases, emphasizing the critical role of imaging features in the differential diagnosis of pulmonary nodules. A comprehensive analysis combining imaging and pathology enables more precise classification of pulmonary nodules of varying nature, providing clearer diagnostic direction for clinical practice.

This study still has some limitations. First, this is a single-center retrospective study with a relatively limited sample size, which may lead to selection bias. The external generalizability of the results still needs to be further verified in multicenter, large-sample studies. Second, the combined detection in this study was based on a parallel testing strategy, which can increase sensitivity but did not involve the construction of a multivariable predictive model, potentially limiting the assessment of interactions among the individual parameters. In addition, this study did not conduct a long-term follow-up analysis and was unable to assess the impact of different diagnostic strategies on patient prognosis. Future studies should expand the sample size and incorporate multivariable analyses and prospective designs to further enhance the stability and clinical utility of the findings. Furthermore, external validation was not performed in the present study, and the cutoffs for tumor markers were based on manufacturer or conventional reference ranges. Future research may explore optimized thresholds more suitable for the SPN population.

## 5 Conclusion

This study demonstrates that the combination of serum tumor markers (CEA, CA-125, CYFRA21-1, NSE) with HRCT imaging features significantly improves the sensitivity and accuracy of distinguishing benign from malignant SPNs. The combined detection strategy demonstrates a high AUC value, indicating high clinical application value in assessing the malignancy risk of pulmonary nodules and providing a reliable basis for early diagnosis and clinical decision-making.

## Data Availability

The original contributions presented in the study are included in the article/supplementary material, further inquiries can be directed to the corresponding author.
